# Safety and efficacy of apatinib in combination treatment versus apatinib as second-line treatment for advanced gastric cancer

**DOI:** 10.3389/fonc.2025.1587069

**Published:** 2025-04-30

**Authors:** Zhang Han, Zhu Yuanzeng, Wu Gang, Sun Peichun

**Affiliations:** The Gastrointestinal Surgery Department, Henan Provincial People’s Hospital, Zhengzhou, China

**Keywords:** gastric cancer, apatinib, combination treatment, efficacy, safety

## Abstract

**Background:**

Apatinib is a systemic therapeutic agent for advanced gastric adenocarcinoma (GAC) and gastroesophageal junction adenocarcinoma (GEJA). Its efficacy can be enhanced by applying it as a combination therapy, but the evidence supporting its combination application as a second-line treatment is not well documented. In the current study, we aimed to assess the efficacy and safety profile of apatinib, both as a monotherapy and in combination regimens, for second-line treatment of GAC and GEJA in real-world settings.

**Methods:**

In this retrospective cohort analysis, we analyzed clinical data from 96 patients with advanced GAC or GEJA who received second-line apatinib monotherapy or combination therapy. Cox regression analysis was performed to identify prognostic factors influencing clinical outcomes of different treatment approaches (apatinib combination with other drugs).

**Results:**

The results indicated that the overall objective response rate (ORR) and disease control rate (DCR) for second-line apatinib therapy were 19.8% and 31.3%, respectively. The median progression-free survival (mPFS) was 4.8 months (95% CI: 4.3-6.2m), while the median overall survival (mOS) was 10.3 months (95% CI: 8.9-12.4m). Multivariable Cox regression analysis identified gender, liver metastasis, and peritoneal metastasis as independent predictors of inferior PFS and OS outcomes. In terms of safety, the primary adverse reactions included myelosuppression, elevated AST and ALT levels, hypertension, hand-foot syndrome, hyperbilirubinemia, proteinuria, fatigue, and vomiting, with a low incidence of grade 3–4 toxicities.

**Conclusions:**

Apatinib-based combination therapy significantly enhances both progression-free survival and overall survival in patients with advanced gastric cancer when compared to monotherapy, while also demonstrating a safe and reliable profile.

## Introduction

Gastric cancer is one of the most prevalent malignant tumors in Asian countries, particularly in China, where its epidemiology may be closely linked to dietary patterns and Helicobacter pylori (HP) infection (1, 2). According to the 2022 Global Cancer Observatory (GLOBOCAN) statistics, approximately 350, 000 new cases of gastric cancer are diagnosed annually, and around 260, 000 cases die with this disease each year (3). However, most early-stage asymptomatic gastric cancer often remains undiagnosed or misdiagnosed, which contributes to therapeutic failure and a high mortality rate (4). Notably, while the incidence of age-standardized incidence rate (ASIR) with gastric adenocarcinoma (GAC) has declined by about 2.3% in recent years, the incidence of gastroesophageal junction adenocarcinoma (GEJA) has shown an annual increase of 1.8% (5, 6). Contemporary therapeutic advances, including cytotoxic chemotherapy, molecularly targeted agents, and immune checkpoint inhibitors, performed a significant decrease in gastric cancer-related mortality (7, 8). Over 70% of patients are found with local advanced or metastatic disease (TNM stage III–IV) disease at the time of initial diagnosis, with a 5-year survival rate remaining alarmingly low at less than 30% (9). Recently, the progress in biomarker-guided immunotherapy and the identification of novel therapeutic targets agents (e.g., CLDN18.2 inhibitors) have significantly enhanced GC patient prognosis in first-line settings (10, 11). Nevertheless, optimizing treatment strategies and precisely identifying patient groups that would benefit from these therapies pose ongoing challenges for future research. Comprehensive treatment based on chemotherapy is considered crucial modality for prolonging the survival of gastric cancer patients (12, 13). While improvements in survival rates have been observed, most patients still experience relapse or develop drug resistance, ultimately resulting in disease progression (14, 15). These limitations underscore the critical requirements for innovative therapeutic approaches integrating multimodal strategies.

Anti-angiogenic therapies selectively inhibit tumor vascular endothelial growth factor (VEGF) signaling pathways and suppress tumor neovascularization and microenvironment remodeling, this mechanistic approach had been shown clinical efficacy across multiple solid tumors (16). The use of anti-angiogenic drugs has also been extensively investigated in the treatment of gastric adenocarcinoma (GAC) and gastroesophageal junction adenocarcinoma (GEJA) (17). Ramucirumab, a monoclonal antibody targeting the VEGF receptor-2, has been approved by the U.S. Food and Drug Administration (FDA) and has established a benchmark for second-line therapy for the treatment of advanced GAC and GEJA (18). Apatinib, an oral tyrosine kinase inhibitor, has been widely used for treating different tumors (19, 20). In 2014, Apatinib was approved in China for treating late-stage GC patients, which showed promising result in phase III trials (AHELP study: mOS 6.5 vs 4.7 months, HR=0.67, P<0.001) (21). In recent years, the combination of apatinib with chemotherapy for advanced-stage GC treatment has garned significant clinical interest (22). Emerging evidence from multicenter observational studies on apatinib in China found that apatinib exhibited satisfactory efficacy and safety in the real-world management of advanced or metastatic gastric cancer (23). Combining apatinib with docetaxel demonstrated superior antitumor activity and manageable toxicity compared to docetaxel monotherapy as a second-line treatment for patients with advanced gastric cancer (24). However, systematic on the clinical efficacy and long-term safety profiles associated with apatinib-based combinations in the treatment of advanced gastric cancer remains incomplete.

To evaluate the real-world efficacy and safety of apatinib combined with chemotherapy or immunotherapy as a second-line treatment for advanced gastric cancer, we retrospectively analyzed 96 patients diagnosed with advanced gastric cancer. Additionally, we compared survival outcomes and toxicity profiles between therapeutic approaches, and identified clinical risk predictors factors of progression free survival through multivariate regression modeling. The findings of our study will help to optimize treatment options available for advanced gastric cancer.

## Materials and methods

### Patients

In this study, we retrospectively analyzed patients with advanced metastatic gastric cancer between April 2022 and October 2024 at the Henan Provincial People’s Hospital of Zhengzhou University. The inclusion criteria for this study were as follows: (1) Aged ≥18 years and <80 years irrespective of gender; (2) Histopathologically confirmed diagnosis of gastric adenocarcinoma; (3) TNM stage IIIB to IV (AJCC 8th edition), with computed tomography (CT) or magnetic resonance imaging (MRI) examinations confirming at least one measurable per response evaluation criteria in solid tumors (RECIST, version 1.1); (4) Availability of complete basic information, including laboratory tests, pathology reports, and imaging data; (5) Eastern Cooperative Oncology Group (ECOG) performance status scores ranging from 0 to 2 (fully active to ambulatory capable of self-care); (6) Normal functioning of major organ systems; (7) Previous failure of conventional first-line chemotherapy. The exclusion criteria were: (1) Pregnant or lactating women (verified by serum β-hCG confirmed in premenopausal women); (2) Patients with hypertension that cannot be controlled to normal levels through antihypertensive treatment; (3) Patients with heart failure or severe liver or kidney dysfunction; (4) Patients with abnormal coagulation function or active bleeding; (5) History of solid organ or bone marrow transplantation; (6) Patients with autoimmune diseases requiring systemic immunosuppressive treatment (prednisone >10 mg/day or equivalent); (7) Patients who discontinue medication for financial reasons; (8) Patients with concurrent primary tumors. The study has been approved by the Ethics Committee of Henan Provincial People’s Hospital (Ethics No. 2022-309-032), and all patients or their respective families provided informed consent.

### Treatment

All patients were administered the patients were categorized into two groups based on therapeutic strategies: the apatinib monotherapy treatment group (43 cases, 250–850 mg/day), and the apatinib combination treatment group (53 cases). The chemotherapy agents utilized in this study primarily included platinum compounds, fluorouracil, taxanes (administered as single agents, in doublet or triplet combinations), and irinotecan. The immunotherapeutic agents comprised camrelizumab, sintilimab, toripalimab, and nivolumab, among others. Evaluation of treatment effects and adverse events, objective evaluation of treatment effects involved conducting CT imaging examinations every six weeks.

### Efficacy and safety

The evaluations were performed according to the solid tumor efficacy evaluation criteria, classifying responses into complete response (CR), partial response (PR), stable disease (SD), and progressive disease (PD). The objective response rate (ORR, %) was calculated as (CR + PR)/total number of cases × 100%, while the disease control rate (DCR, %) was determined by (CR + PR + SD)/total number of cases × 100%. Progression-free survival (PFS) was defined as the duration from the initiation of treatment to the last follow-up or death prior to disease progression, whereas overall survival (OS) is defined as the time from the start of treatment to death or the last follow-up.

### Tumor marker analysis

Peripheral blood samples (4 mL) were collected in EDTA anticoagulant tubes from all patients, then centrifuged samples at 3000 rpm for 10 min and serum samples were retained. The levels of carbohydrate antigen 19-9 (CA19-9), carcinoembryonic antigen (CEA), carbohydrate antigen 72-4 (CA72-4) and carbohydrate antigen 125 (CA125) were detected by electrochemical luminescence (Roche Cobas e601 analyzer).

### Statistical analysis

Statistical analysis was conducted using SPSS version 26.0. Categorical variables were expressed as frequencies (%) and with the χ2 test or Fisher’s exact test utilized for comparison. Survival analysis was performed using the Kaplan-Meier method, and GraphPad version 8.0 was used to generate survival curves, with two-tailed P<0.05 denoting statistical significance. Prognostic factors were identified through univariate and multivariate Cox proportional hazards regression (variables with P<0.10 in univariate analysis entered multivariate modeling).

## Results

### Baseline characteristics of patients

A total of 96 patients with histologically confirmed advanced gastric adenocarcinoma treated with apatinib (monotherapy or combination therapy) from April 2022 to October 2024 were included in the study. In the total population, the demographic analysis revealed a median age of 59.0 years, with 31.3% (30/96) predominantly female, and 36.4% (35/96) presented with more than two metastatic lesions. The detailed clinical characteristics of the patients are summarized in **Table 1**. There was no difference in baseline characteristics between apatinib monotherapy and combination groups. Regarding key baseline characteristics, the monotherapy (n=43) and combination therapy (n=53) cohorts were balanced including age, gender, ECOG performance status, histologic differentiation, primary tumor, history of surgery, and metastatic patterns.

**Table 1:** Baseline characteristics of all enrolled patients.

Variables	Monotherapy group (N=43)	Combined group (N=53)	χ2	P-value
Age	59.4	55.8		
Gender			0.04	0.84
Female	13 (30.2%)	17 (32.1%)		
Male	30 (69.8%)	36 (67.9%)		
ECOG			1.76	0.18
0	15 (34.9%)	12 (22.6%)		
1	28 (65.1%)	41 (77.4%)		
Histologic differentiation			0.35	0.55
Moderately	4 (9.3%)	7 (13.2%)		
Poorly	39 (90.7%)	46 (86.8%)		
Primary tumor			0.98	0.32
Gastroesophageal junction	3 (7.0%)	7 (13.2%)		
Gastric	40 (93.0%)	46 (86.8%)		
History of surgery			0.01	0.97
Yes	12 (27.9%)	15 (28.3%)		
No	31 (72.1%)	38 (71.7%)		
Peritoneal metastasis			0.02	0.88
Yes	6 (14.0%)	8 (15.1%)		
No	37 (86.0%)	45 (84.9%)		
Liver metastasis			0.03	0.87
Yes	7 (16.3%)	8 (15.1%)		
No	36 (83.7%)	45 (84.9%)		
Number of metastatic lesions			0.02	0.89
≤2	27 (62.8%)	34 (64.2%)		
>2	16 (37.2%)	19 35.8%)		

### Overall clinical benefit

The most common initial dose of Apatinib was 500 mg/day (51/96, 53.1%), followed by 250 mg/day (36/96, 37.5%). Comparative analysis between apatinib monotherapy and combination regimens revealed distinct clinical outcomes: 2 patients (4.7%) achieved complete response (CR) and 4 patients (9.3%) achieved partial response (PR) in the monotherapy cohort; the combination cohort exhibited a numerically higher objective response rate: 2 patients (3.8%) achieved CR and 11 patients (20.7%) achieved PR. The overall response rates (ORR) were 14.0% (6/43, 95 %CI: 6.6%-27.3%) and 24.5% (13/53, 95 %CI: 14.9%-37.6%), respectively, which did not reach statistical significance (χ²=1.67, P = 0.196). Similarly, the disease control rates (DCR) were recorded at 23.2% (10/43, 95 %CI: 13.2%-37.7%) and 37.7% (20/53, 95 %CI: 25.9%-51.2%), respectively, also showed no statistical significance (χ²=2.317, P = 0.128).

**Figure 1:**
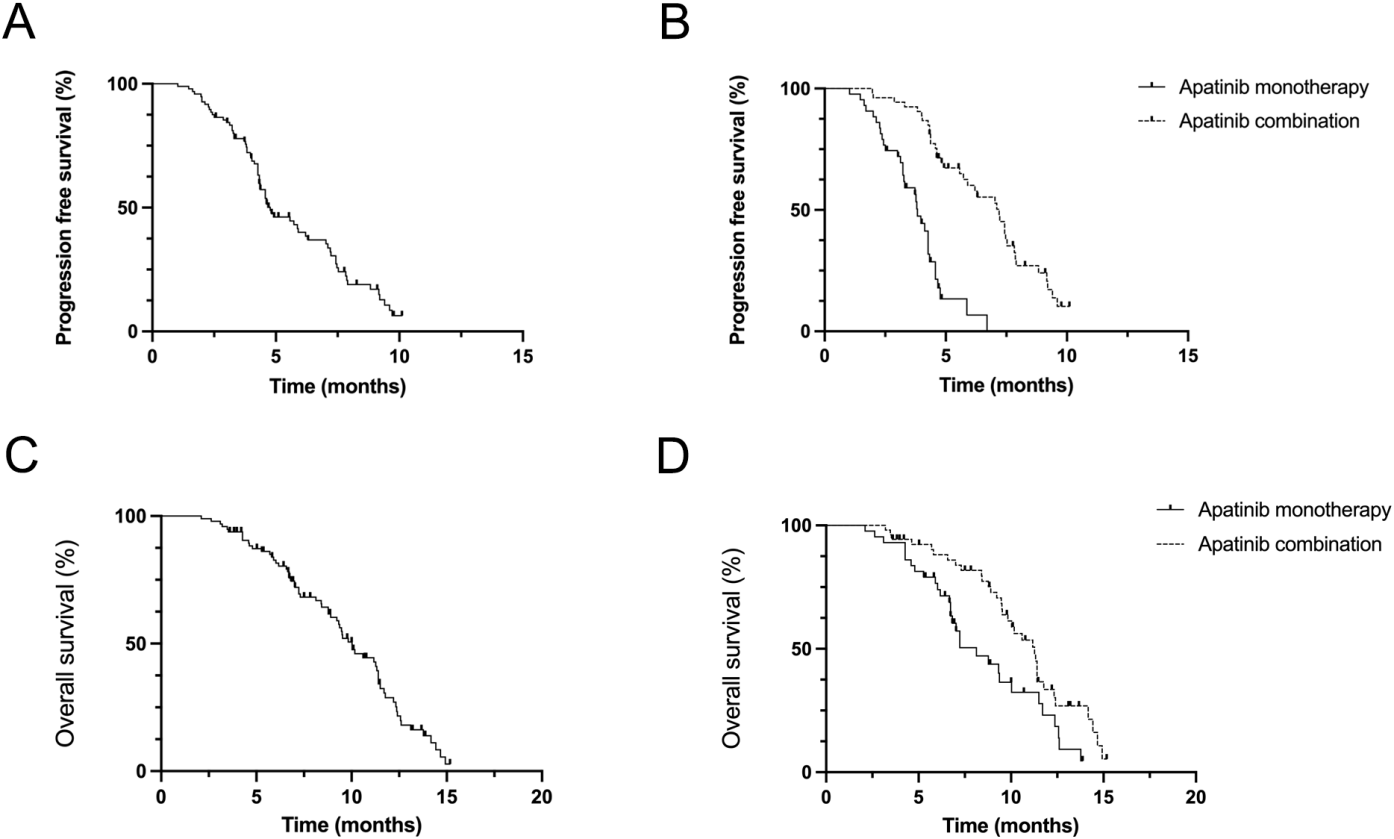
Efficacy evaluation of second-line with apatinib monotherapy and combination treatment **(A, B)** Kaplan–Meier analysis of progression-free survival in patients treated with apatinib monotherapy and combination treatment. **(C, D)** Kaplan–Meier analysis of overall survival in patients treated with an apatinib monotherapy and combination treatment.

**Figure 2:**
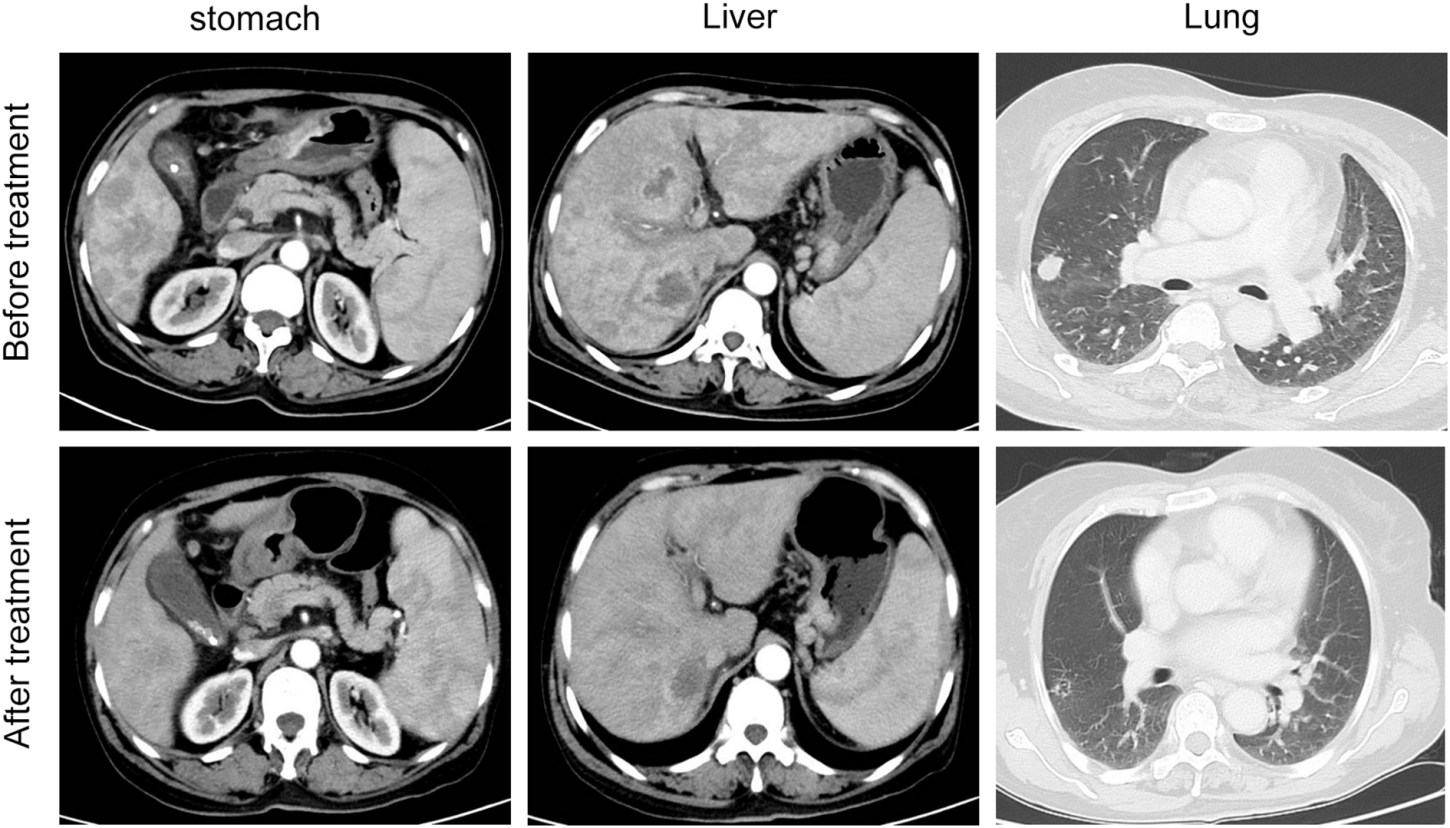
The clinical course of the patient in the apatinib combination cohort patient with liver and lung metastases before and after treatment.

The overall progression free survival (PFS) of the 96 patients who received apatinib treatment was 4.8 months (95% CI: 4.3-6.2m). The median PFS (mPFS) for the apatinib monotherapy and combination treatment regimens were 3.8 months (95% CI: 3.2-4.3m) and 7.2 months (95% CI: 5.7-7.5m), respectively (**Figures 1A, B**). The overall survival (PFS) of the 96 patients who received apatinib treatment was 10.3 months (95% CI: 8.9-12.4m), the median OS (mOS) for the apatinib monotherapy and combination treatment regimens were 8.1 months (95% CI: 6.7-11.0m) and 11.3 months (95% CI: 8.5-12.7m), respectively (**Figures 1C, D**). The magnitude of survival benefit and highly significant P-values (both P<0.001) results indicated that the superiority of combination therapy in prolonging both PFS and OS for advanced gastric cancer patients undergoing second-line treatment. One patient of gastric cancer with liver and lung metastases who performed apatinib combined with sintilimab showed significant tumor response after treatment (**Figure 2**). The PFS of the patient was 5.5 months.

### Tumor biomarkers level in patients

Peripheral venous blood samples (4 mL) were obtained from all patients and centrifuged at 3000 rpm for 10 min. Before treatment, the biomarker levels of CEA (133.87 ± 20.69 vs. 137.11 ± 19.93, t=0.779, P=0.438), CA19-9 (203.44 ± 35.29 vs.211.27 ± 34.55, t=1.094, P=0.277), CA72-4 (124.38 ± 29.54 vs. 133.43 ± 27.45, t=1.552, P=0.124) and CA125 (60.27 ± 12.44 vs. 57.71 ± 11.57, t=1.042, P=0.300) were not significantly different among the two groups in pretreatment biomarker levels. After treatment, the biomarker levels of CEA (80.04 ± 13.77 vs. 73.78 ± 11.13, t=2.464, P=0.016), CA19-9 (100.15 ± 20.25 vs. 90.25 ± 17.31, t=2.582, P=0.011), CA72-4 (77.53 ± 17.69 vs. 65.14 ± 13.51, t=3.890, P=0.000) and CA125 levels (33.79 ± 6.25 vs. 25.49 ± 4.41, t=7.614, P=0.000) in monotherapy and combination group, respectively, were significantly reduced (P<0.05, paired t-test), the combination therapy cohort showed better effects compared with monotherapy (**Table 2**).

**Table 2:** Analysis of tumor biomarkers between the monotherapy treatment group and combination group ( $\bar{\text{x}}$ ±s).

Group	CEA (ng/mL)		CA19-9 (U/mL)		CA72-4 (U/mL)		CA125 (U/mL)	
	Before	After	Before	After	Before	After	Before	After
Monotherapy (n=43)	133.87±20.69	80.04±13.77^*^	203.44±35.29	100.15±20.25^*^	124.38±29.54	77.53±17.69^*^	60.27±12.44	33.79±6.25^*^
Combination (n=53)	137.11±19.93	73.78±11.13^*#^	211.27±34.55	90.25±17.31^*#^	133.43±27.45	65.14±13.51^*#^	57.71±11.57	25.49±4.41^*#^
t	0.779	2.464	1.094	2.582	1.552	3.890	1.042	7.614
P	0.438	0.016	0.277	0.011	0.124	0.000	0.299	0.000

^*^P<0.05; ^#^P<0.05.

### Analysis of influencing factors for mPFS and mOS

To identify prognostic determinants of PFS and OS in apatinib-treated patients, univariate and multivariate Cox regression models were constructed to understand the factors potentially in apatinib treatment. Univariate Cox regression analysis revealed that gender (male vs female) (P=0.020, hazard ratio (HR)=8.257), the presence of liver metastasis (with vs without) (P=0.001, HR=6.560) and peritoneal metastasis (with vs without) (P=0.040, HR=2.975) were significantly associated with poorer progression-free survival (PFS). Multivariate Cox regression analysis confirmed male as an independent predictor of PFS deterioration (P=0.005, HR=20.431), hepatic metastasis emerged as the predominant risk factor (P=0.005, HR=11.256), and peritoneal metastasis was confirmed predominant risk factor (P=0.070, HR=12.221), these factors were independently linked to worsening PFS (**Table 3**). Regarding OS, hepatic metastasis (P=0.001, HR=6.438) and peritoneal metastasis (P=0.019, HR=3.544) were identified as independent predictors associated with OS deterioration. The final multivariate Cox regression model validated analysis that hepatic metastasis (P=0.001, HR=15.037) and peritoneal metastasis (P=0.001, HR=8.874) are independently associated with OS deterioration (**Table 4**).

**Table 3:** Univariate analysis and multivariate analysis for influencing factor of second line treatment progression free survival.

Factor	Univariate analysis			Multivariate analysis		
	HR	95%CI	P	HR	95%CI	P
Gender (Male vs Female)	8.257	1.549-11.142	0.020	20.431	3.108-41.371	0.005
Age (≥60 years old vs<60 years)	1.142	0.306-2.383	0.491			
ECOG PS (1 vs 0)	1.371	0.042-3.392	0.440			
Primary tumor (Gastroesophageal junction vs. Gastric)	1.352	0.375-2.657	0.598			
History of surgery (Yes vs. No)	0.889	0.474-1.669	0.715			
Metastasis number (≥2 vs<2)	1.453	0.503-3.007	0.714			
Hepatic metastasis (Yes vs. No)	6.560	3.431-10.893	0.001	11.256	2.966-22.711	0.005
Peritoneal metastasis (Yes vs. No)	2.975	0.947-5.863	0.040	12.221	3.172-25.360	0.070
Histologic differentiation (Poorly vs. Moderately)	2.572	1.118-5.671	0.086			
Combination treatment (Yes vs. No)	0.236	0.083-0.724	0.001	0.147	0.035-0.699	0.007

**Table 4:** Univariate analysis and multivariate analysis for influencing factor of second line treatment overall survival.

Factor	Univariate analysis			Multivariate analysis			
	HR	95%CI	P	HR	95%CI		P
Gender (Male vs Female)	1.139	0.247-3.110	0.320				
Age (≥60 years old vs<60 years)	1.103	0.372-2.446	0.441				
ECOG PS (1 vs 0)	3.368	0.882-5.317	0.351				
Primary tumor (Gastroesophageal junction vs. Gastric)	1.052	0.261-2.447	0.598				
History of surgery (Yes vs. No)	0.811	0.135-1.312	0.315				
Metastasis number (≥2 vs<2)	1.576	0.451-2.016	0.459				
Hepatic metastasis (Yes vs. No)	6.438	2.775-20.811	0.001	15.037	3.377-30.561		0.001
Peritoneal metastasis (Yes vs. No)	3.544	1.567-7.847	0.019	8.874	2.396-25.487		0.001
Histologic differentiation (Poorly vs. Moderately)	2.302	0.438-5.451	0.139				

### Adverse events

Currently, 22.9% of patients dose reduced due to adverse events, while 72.1% did not reduce dose of apatinib. Adverse event reporting may underestimate actual clinical incidence. The incidence of any grade includes Myelosuppression, AST elevation, ALT elevation, Hypertension, Hand-foot syndrome, Elevated bilirubin, Proteinuria, Fatigue, Vomiting. For the patients treated with apatinib monotherapy, the adverse events above grade 3 are mainly myelosuppression (9.3%), AST elevation (4.6%), ALT elevation (4.6%), hand-foot syndrome (7.0%), and elevated bilirubin (2.3%). For patients treated with the Apatinib combination, grade 3 or higher adverse events were observed in all of the adverse events collected (**Table 5**).

**Table 5:** Treatment related adverse events of all the patients.

Adverse event	Monotherapy group (N=43)		Combined group (N=53)	
	Grade 1~2, n (%)	Grade 3~4, n (%)	Grade 1~2, n (%)	Grade 3~4, n (%)
Myelosuppression	14 (32.6%)	4 (9.3%)	20 (37.7%)	5 (9.4%)
AST elevation	6 (13.9%)	2 (4.6%)	12 (22.6%)	3 (5.7%)
ALT elevation	6 (13.9%)	2 (4.6%)	12 (22.6%)	3 (5.7%)
Hypertension	5 (11.6%)	0	16 (30.1%)	2 (3.8%)
Hand-foot syndrome	4 (9.3%)	3 (7.0%)	13 (24.5%)	3 (5.7%)
Elevated bilirubin	4 (9.3%)	1 (2.3%)	8 (15.1%)	3 (5.7%)
Proteinuria	13 (30.2%)	0	14 (26.4%)	2 (3.8%)
Fatigue	4 (9.3%)	0	8 (15.1%)	1 (1.9%)
Vomiting	9 (20.9%)	0	8 (15.1%)	2 (3.8%)

## Discussion

The annual incidence rate of gastric cancer in age-standardized incidence rates (ASIR) is notably progressive and represents a major global health burden (3). This disease predominantly affects individuals aged 40 to 70 years, with 1.5- to 2-fold higher incidence in males (25). In recent years, emerging studies have shown an alarming rise in GC occurrence in young individuals, attributed to rising occupational stress, high sodium intake and processed food consumption (26). Furthermore, geographical heterogeneity is particularly pronounced, the incidence in southern China (Guangdong: 11.2/100,000) is markedly lower compared to that in the northwestern (Gansu: 35.6/100,000) and eastern coastal regions (27). Early detection through endoscopic surveillance plays a crucial role in enhancing the prognosis of gastric cancer patients (28). However, the insidious nature of early symptoms often results in diagnoses at advanced stages, where patients present with melena, hematemesis, malnutrition, and pain, severely compromising their quality of life (4, 6). While the precise pathogenesis of gastric adenocarcinoma remains incompletely elucidated, it is commonly associated with multifactorial pathogenesis involving premalignant progression, immune dysregulation, and environmental exposures (29). Notably, individuals with a family history of gastric cancer exhibit an incidence rate two to three times higher particularly in families with germline CDH1 mutations (30). Surgical resection is the preferred treatment option for localized gastric cancer, it cannot be confidently applied on advanced-stage GC patients, and a notable recurrence rate post-surgery is observed (27). Recent studies indicate that the combination of targeted therapies and radiotherapy yields satisfactory outcomes for patients with advanced gastric cancer (31).

As a highly selective vascular endothelial growth factor receptor-2 inhibitor, apatinib suppresses tumor angiogenesis by potently inhibiting VEGF-mediated signaling pathways critical for tumor neovascularization, enabling it clinical efficacy in patients with recurrent or progressive advanced gastric adenocarcinoma (32, 33). Furthermore, the tumor vascular normalization can facilitate the accumulation of immune cells and enhance immune functionality, particularly CD8+ T lymphocytes and dendritic cells (34). This immunomodulatory effect creates a self-reinforcing cycle in immune cells and blood vessels (34). This reciprocal mechanism underlies the rationale for combining anti-angiogenic drugs with immune checkpoint inhibitors, providing a translational foundation for treating malignant tumors.

A prospective clinical trial investigated the combination of apatinib and platinum-based chemotherapy as a second-line treatment for advanced gastric cancer and documented a mPFS of 3.5 months (35). Camrelizumab-apatinib combination as a second-line treatment achieved an mOS of 12.1 months for advanced gastric cancer adenocarcinoma cohorts (23). The observed 5-year survival rates for patients with GAC or GEJA remain suboptimal, spanning 5-30% across clinical series (27, 28), partly a phenomenon attributed to the paucity of treatment options available following first-line treatment failure (9). Notably, pharmacotherapy analyses reveal that apatinib plus chemotherapy as a second-line therapy setting for GAC or GEJA patients results in a median PFS of 3.06 and OS of 6.51 months (22). Among patients undergoing second-line advanced GAC treated with apatinib plus S-1, the median PFS and OS were reported as 143.1 days and 211.6 days, respectively (36). In the present phase II study, patients with advanced GAC or GEJA receiving second-line apatinib plus inotecan exhibited median PFS and OS of 4.5 and 11.7 months, respectively (37).

Real-world studies from our institution indicate that patients’ PFS was comparable to or exceeded previous studies, irrespective of whether patients received monotherapy or combination therapy. This is possibly linked to optimized apatinib dosage schedules and prognostic profiles, younger patient age at diagnosis, lower ECOG performance status scores, and oligometastatic disease patterns. Furthermore, multivariable Cox regression analysis revealed male, hepatic metastases, and peritoneal metastasis as independent prognostic determinants for deteriorating PFS or OS. Importantly, these high-risk subgroups patients necessitate targeted clinical management rather than exclusion from clinical research. In our study, we also analyzed tumor markers (CEA, CA19-9, CA72-4, CA125) in both monotherapy and combination group before and after treatment. Our findings suggest that combinations test of CEA, CA19-9, CA72–4 and CA125 is an effective approach for treatment evaluation both prior to and following therapeutic interventions.

Existing evidence delineates the primary apatinib-associated treatment-emergent adverse events (TEAEs) including myelosuppression such as leukopenia, granulocytopenia, and thrombocytopenia, as well as non-hematological complications such as proteinuria, hypertension, hand-foot syndrome, fatigue, and hoarseness (21, 38). Apatinib combination regimens incorporating agents require vigilant monitoring for overlapping toxicities, such as chemotherapy and immune checkpoint inhibitors, it is essential to consider the adverse reactions associated with chemotherapy, including fatigue, nausea and vomiting, elevated transaminases, and diarrhea.

Our pharmacovigilance data confirm the overall clinical manageability of these toxicities through protocol-specified dosage modifications and supportive interventions.

However, our study has three principal limitations. Firstly, the sample size is not sufficiently large; the modest cohort size warrants multicenter validation to enhance statistical power for real-world outcome assessments. Secondly, the efficacy of various combination therapies warrants further analysis. Third, comparative effectiveness analyses across heterogeneous therapeutic combinations require systematic pharmacogenomic profiling.

## Conclusion

Apatinib-based combination therapy demonstrates better efficacy than monotherapy in second-line advanced gastric cancer. Our study achieved statistically significant improvements in both progression-free survival and overall survival, with manageable toxicity profiles under standardized toxicity mitigation protocols. Larger sample sizes are required to further validate efficacy of second-line apatinib-based combination therapy, potentially supporting evidence first-line treatment in patients with advanced GAC or GEJA.

## Data Availability

The original contributions presented in the study are included in the article/supplementary material. Further inquiries can be directed to the corresponding author.
